# *N,N*-Dimethylaminoxy Carbonyl, a Polar Protecting Group for Efficient Peptide Synthesis

**DOI:** 10.3389/fchem.2019.00173

**Published:** 2019-03-28

**Authors:** Ryo Okamoto, Emiko Ono, Masayuki Izumi, Yasuhiro Kajihara

**Affiliations:** Department of Chemistry, Osaka University, Toyonaka, Japan

**Keywords:** *N,N*-dimethylaminoxy carbonyl, Dmaoc, Dbz, peptide coupling, solid phase peptide synthesis, chemical ligation, thioester, protecting group

## Abstract

Peptide coupling with minimal protection is one of the desired methods for the synthesis of peptides and proteins. To achieve regioselective amide bond formation, side chain protection is often essential; however, protecting groups potentially diminish peptide solubility and render the polar polyamide chain amphipathic due to their apolar nature. In this manuscript, we describe a new protecting group, *N,N*-dimethylaminoxy carbonyl (Dmaoc), and its use in peptide coupling reactions. The Dmaoc group has a relatively polar character compared to the Boc group, which is a conventional protecting group for the *N*^ε^-amine of Lys residues. This polar protecting group is removable by reduction in the buffer containing (±)-dithiothreitol (DTT). Furthermore, the Dmaoc group proved compatible with peptide ligation strategies featuring the activation of *N*-acyl diaminobenzamides (Dbz) with sodium nitrate to generate the respective benzotriazole leaving group. The Dmaoc/Dbz strategy described in this manuscript provides a new method for the chemical synthesis of peptides.

## Introduction

Peptide coupling reactions are essential for the chemical synthesis of polypeptides and proteins (Kricheldorf, 2006; Kent, 2017). Considerable efforts have been devoted to the development of chemical ligation for the chemoselective coupling of unprotected peptides. Many proteins have been synthesized using a variety of chemical ligation techniques, including native chemical ligation (Bode, 2011, 2017; Jin and Li, 2018; Liu and Li, 2018). These methodologies are generally based on site-specificity, which limits the amino acid sites for ligations. Consequently, chemical ligation often requires the preparation of suitable building blocks for peptide coupling at desired sites, depending on the amino acid sequence of target polypeptides.

Strategies are desired for the coupling of peptide fragments possessing diverse chemical structures using minimal functional group protection (Aimoto, 1999; Hojo, 2014). To achieve regioselective amide bond formation, side chain protection is often essential; however, many protecting groups diminish peptide solubility and render the polar polyamide chain amphipathic due to their nonpolar nature. A strategy involving minimal use of protection on the *N*^ε^-amine of Lys residues and the *N*-terminal α-amine may circumvent the solubility problem and avoid undesirable amide bond formation. The utility of the minimum protection strategy for peptide coupling was demonstrated in the chemical synthesis of proteins using the thioester leaving group (Aimoto, 1999). However, this strategy used Boc protection on the *N*^ε^-amine of Lys, which increases the risk of poor solubility, depending on the number of protecting groups in the target peptide sequence. The isonicotinyloxycarbonyl (iNoc) group has been used to circumvent the intrinsic low solubility of protected peptides (Veber et al., 1977). Recently, the protection of the *N*^ε^-amine of Lys residues with the iNoc group has also demonstrated its utility during peptide synthesis (Asahina et al., 2015). These reports suggest the potential of polar protecting groups for the chemical synthesis of peptides. However, much effort has not been paid to the development of new polar protecting groups that are stable but efficiently removable under mild reaction conditions.

Herein, we present a polar protecting group *N,N*-dimethylaminooxy carbonyl (Dmaoc) for the *N*^ε^-amine of Lys and its use in peptide synthesis. This protecting group can be removed by reduction in a buffer containing thiol. The Dmaoc group proved compatible with peptide ligation strategies featuring the activation of *N*-acyl diaminobenzamides (Dbz) with sodium nitrate to generate the respective benzotriazole (Bt) leaving group (Wang et al., 2015; Weidmann et al., 2016), as further demonstrated by the synthesis of Sunflower trypsin inhibitor.

## Results and Discussion

**Figure 1:**
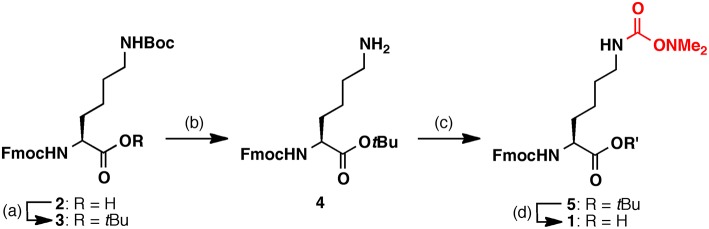
Synthesis of Fmoc-Lys(Dmaoc)-OH **1**. Reagents and conditions: **(a)** Boron trifluoride diethyl ether complex, *tert*-butyl 2,2,2-trichloroacetimidate; **(b)** TFA, dichrolomethane (y = 86%, over 2 steps); **(c)** *N,N'*-carbonyldiimidazole, *N,N*-dimethyl hydroxylamine hydrochloride (y = 46%); **(d)** 6 M hydrochloric acid, 1,4-dioxane (y = 91%).

Firstly, Fmoc-Lys(Dmaoc)-OH **1** was synthesized from commercially available Fmoc-Lys(Boc)-OH **2** for the preparation of Dmaoc protected peptide by Fmoc solid phase peptide synthesis (SPPS) (Figure 1). The Lys derivative **2** was esterified using *tert*-butyl trichloroacetimidate. The Boc group was removed selectively by treatment with trifluoroacetic acid (TFA)/dichloromethane solution. The Dmaoc group was then incorporated onto the *N*^ε^-amine of Lys of **4** by treatment with *N,N'*-carbonyldiimidazole, followed by the addition of *N,N*-dimethylhydroxylamine. After optimizing the conditions for removing the *t*Bu ester moiety, we found that the treatment of the fully protected Lys derivative **5** with the mixed solution of 6 M HCl and 1,4-dioxane (1: 1 vol/vol) afforded Fmoc-Lys(Dmaoc)-OH **1** in good yield.

In order to perform a peptide coupling reaction, we focused on the Dbz group that was known as a precursor of the Bt group. Thioesters have been utilized as an efficient leaving group for peptide coupling reactions. However, the synthesis of peptide-thioesters is still difficult in Fmoc SPPS, and thus several thioester surrogates have been developed. Recently, the Dawson and Liu groups have independently reported the synthesis of peptide-thioester from peptide-Dbz through the formation of peptide-Bt (Wang et al., 2015; Weidmann et al., 2016). These synthetic methodologies were based on previous studies by Katritzky et al. (1992, 2000, 2004), in which *N*-acyl-Bt was used as an efficient acylating agent. We envisaged that peptide-Bt, prepared from peptide-Dbz, could be used as a *C*-terminal activated peptide for peptide coupling reactions.

**Figure 2:**
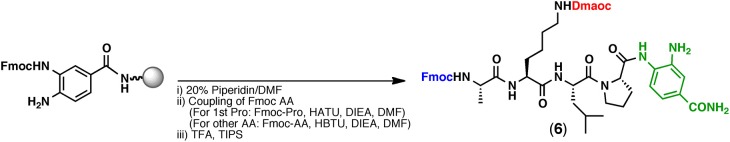
Fmoc SPPS of Dmaoc-protected peptide (**6**).

The Dmaoc-protected peptide having the Dbz group was synthesized by standard Fmoc SPPS (Figure 2). The synthesis was performed on the commercially available Fmoc-Dbz-polystyrene resin, according to the protocol reported by Dawson et al. (Blanco-Canosa and Dawson, 2008). Coupling of Fmoc-amino acids including **1** was performed by using standard coupling reagents such as HATU or HBTU, together with *N,N*-diisopropylethylamine (DIEA) (see Supplementary Material for the detail). Removal of the Fmoc group was also performed under standard conditions using 20% piperidine/*N, N*-dimethylformamide (DMF). After complete assembly, the peptide was detached from the solid support by use of a TFA cocktail. This Fmoc SPPS successfully yielded the Dmaoc-protected peptide-Dbz (peptide(Dmaoc)-Dbz **6**, 66% isolated yield).

**Figure 3:**
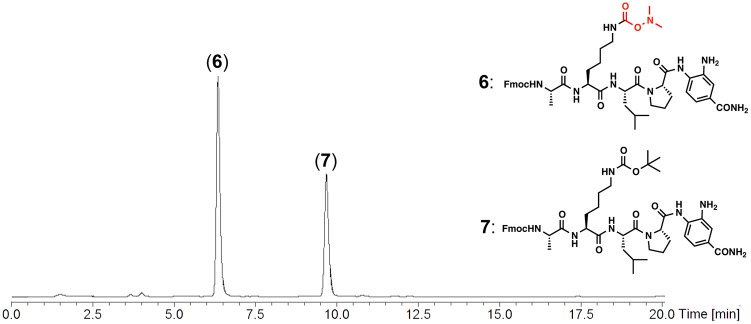
RP-HPLC chromatogram for the analysis of Dmaoc-protected peptide (**6**) and Boc-protected peptide (**7**). Chromatographic separation was performed by using a linear gradient of 0.1% HCOOH aq.: 90% acetonitrile aq. containing 0.09% HCOOH from 60:40 to 30:70 over 20 min at a flow rate of 0.3 mL/min. C4 silica gel with column dimension 2.0 × 150 mm was used for this analysis. The eluent was monitoring by UV-absorbance at 280 nm with on-line ESI-MS.

The Dmaoc group includes the dimethylamino moiety that can be a proton acceptor and this structural property allowed us to anticipate the polar nature of the Dmaoc group. The polarity of the Dmaoc group was compared with the Boc group, a conventional protecting group for the *N*^ε^-amine of Lys. The peptide(Dmaoc)-Dbz **6** and corresponding Boc-protected peptide **7** were subjected to reversed-phase high-performance liquid chromatography (Figure 3). This analysis revealed that the retention time for the peptide(Dmaoc)-Dbz **6** was shorter than that of the peptide(Boc)-Dbz **7**. This result indicates that the Dmaoc group has essentially higher polarity than that of Boc group.

**Figure 4:**
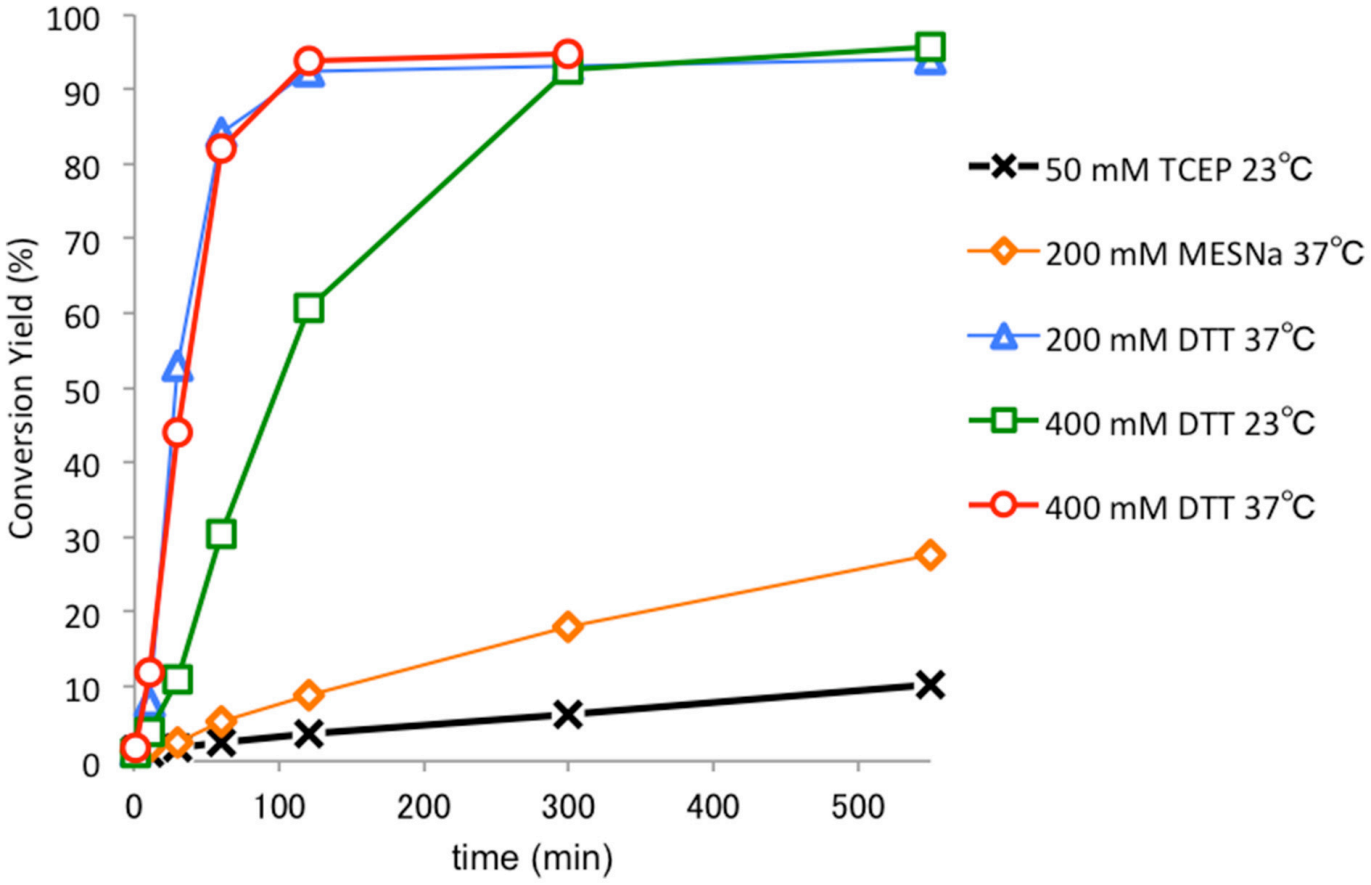
Investigation of the reaction rate for the removal of Dmaoc group of peptide **6**. The reaction was performed using 4 mM of the peptide (0.5 mg) in 0.1 M sodium phosphate buffer containing 6 M guanidine hydrochloride and reducing agents at pH 7.0. At each time point, the fraction conversion was determined by HPLC peak area intensities.

We found that the Dmaoc group could be removed under reductive conditions (Figure 4). The removal of the Dmaoc group on peptide **6** was conducted in a buffer at pH 7.0 in the presence of reducing agents such as sodium 2-mercaptosulfonate (MESNa), (±)-dithiothreitol (DTT) or tris(2-carboxyethyl)phosphine (TCEP). Consequently, the Dmaoc group was efficiently removed within a few hours in the presence of DTT. In contrast, the deprotection rate was slow in the presence of MESNa or TCEP, and no deprotection was observed without reducing agents. These results suggested that a suitable reducing potency as well as the nucleophilicity of the thiol group contributed to the removal of Dmaoc group; however, a detailed investigation of the reaction mechanism is still underway.

**Figure 5:**
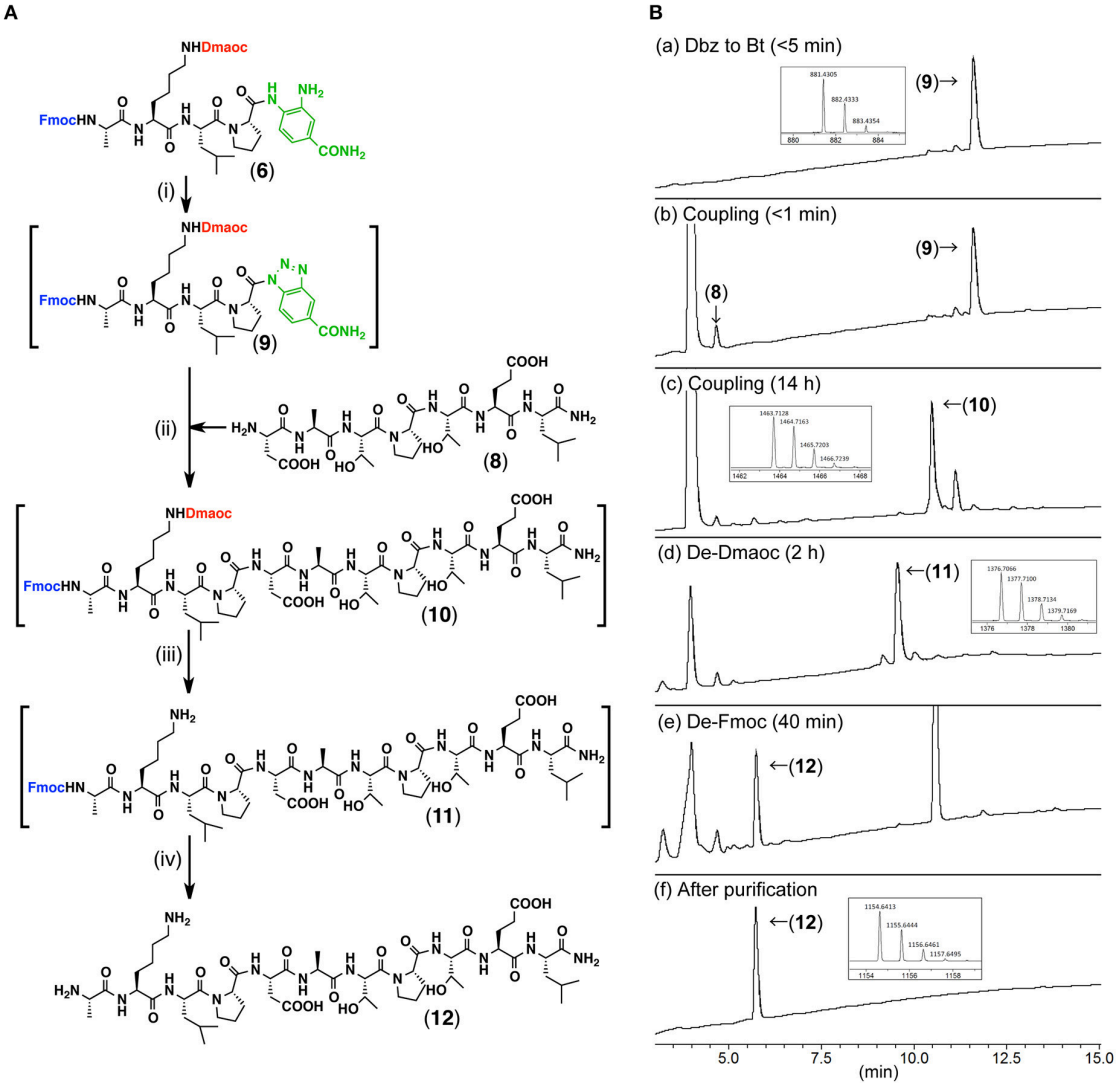
The coupling of the Dmaoc-protected peptide-Dbz **6** and the heptapeptide **8**. **(A)** Reaction scheme for the peptide coupling followed by the deprotections; Reagents and conditions: (i) NaNO_2_, DMF/4 M HCl in 1,4-dioxane (39: 1 vol/vol); (ii) DIEA, HOOBt, DMF; (iii) Ether precipitation then 0.1 M sodium phosphate buffer containing 6 M guanidine-hydrochloride and 0.2 M DTT (pH 7.0) at 37°C; (iv) piperidine. **(B)** LC data for the monitoring of the sequential reactions. Analytical condition: Linear gradient of 0.1% HCOOH aq.: 90% acetonitrile aq. containing 0.09% HCOOH from 90:10 to 30:70 over 15 min at a flow rate of 0.3 ml/min. The eluent was monitoring by UV-absorbance at 218 nm with on-line ESI-MS. Insets are expanded view of [M+H]^+^ ion peak acquired by on-line ESI-HRMS from each UV peak labeled with compound number. m/z calculated mono isotopic [M+H]^+^ for compound **9** C_45_H_57_N_10_${\text{O}}_{9}^{+}$ 881.4304; for compound **10** C_69_H_103_N_14_${\text{O}}_{21}^{+}$ 1463.7417; for compound **11** C_66_H_98_N_13_${\text{O}}_{19}^{+}$ 1376.7096; for compound **12** C_51_H_88_N_13_${\text{O}}_{17}^{+}$ 1154.6416.

We then tested the peptide coupling between the peptide(Dmaoc)-Dbz **6** and the heptapeptide **8** (Figure 5A, Figures S-1,S-4). The Dbz group was converted into Bt by treatment with sodium nitrate (NaNO_2_) in acidic DMF at −17°C (Figure 5Ba). Subsequently, peptide **8**, 3-hydroxy-1,2,3-benzotriazine-4(3*H*)-one (HOOBt) and DIEA were added to initiate the peptide coupling via the conversion of peptide-Bt (**9**) into an active peptide-ester. This reaction gave Dmaoc-protected undecapeptide **10** within 14 h (Figure 5Bc). After separating the crude peptide **10** from DMF by ether precipitation, the resultant material was dissolved in phosphate buffer (pH 7.0) containing DTT to remove the Dmaoc group. This reaction proceeded to completion within 2 h and gave peptide **11** (Figure 5Bd). To remove the *N*-terminal Fmoc group, piperidine was subsequently added to the reaction mixture (Figures 5Be,f). As a result, these sequential reactions efficiently yielded undecapeptide **12** (60% isolated yield over four steps).

Encouraged by the result of the peptide coupling reaction, we conducted the synthesis of a cyclic peptide using the Dmaoc/Dbz strategy. Sunflower trypsin inhibitor (SFTI) is one of the smallest cyclic peptides that has a head-to-tail ring structure consisting of 14 amino acids, including one Lys residue. The chemical synthesis of SFTI has been achieved using native chemical ligation, which requires an *N*-terminal cysteine residue (Daly et al., 2006). In our present research, the Dmaoc/Dbz strategy was applied to the construction of the cyclic-structure of SFTI. We expected that this result would provide us with a new synthetic route for cyclic peptides that do not have Cys residues.

**Figure 6:**
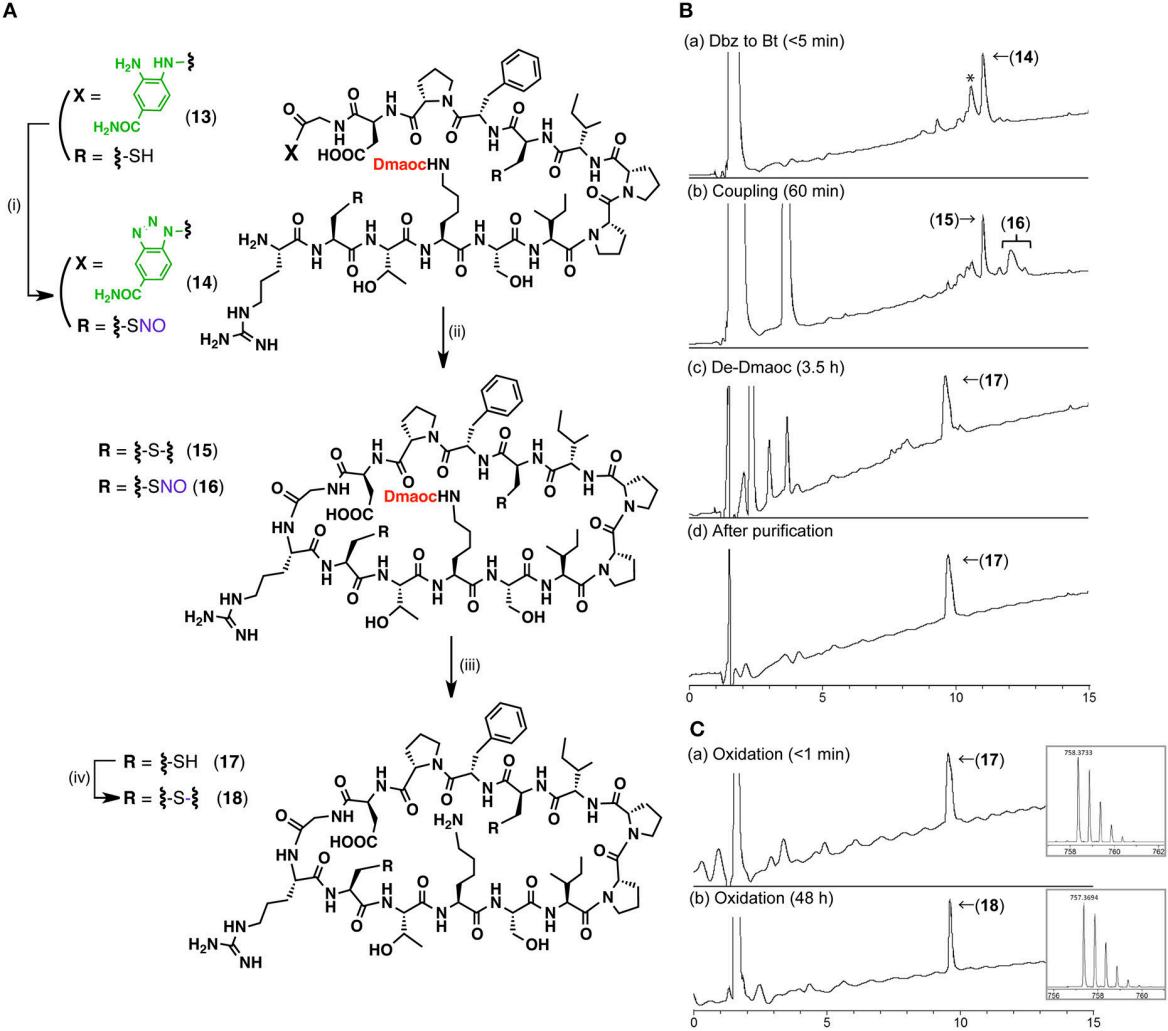
The synthesis of cyclic peptide, SFTI using the Dmaoc protected peptide-Dbz (**13**). **(A)** Reaction scheme for the synthesis of SFTI; Reagents and conditions: (i) NaNO_2_, DMF / 4 M HCl in dioxane (39: 1, vol/vol); (ii) DIEA, HOOBt, DMF; (iii) Ether precipitation then 0.1 M sodium phosphate buffer containing 6 M guanidine-hydrochloride and 0.2 M DTT (pH 7.0) at 37°C; (iv) 20 % DMSO aq. **(B)** LC data for the monitoring of the sequential reactions and **(C)** LC data for the formation of disulfide bond; Analytical condition: Linear gradient of 0.1% HCOOH aq.: 90% acetonitrile aq. containing 0.09% HCOOH from 90:10 to 30:70 over 15 min at a flow rate of 0.3 ml/min. The eluent was monitoring by UV-absorbance at 218 nm with on-line ESI-MS. Peak labeled with asterisk in the chromatogram of **(Ba)**, was corresponding to the hydrolyzed form of peptide **14** judged by on-line MS analysis (see Supplementary Material). This was plausibly afforded in LC-MS analysis and not from reaction mixture, since this product was not observed in the following coupling step; Insets are expanded view of [M+H]^+^ ion peak acquired by on-line ESI-HRMS from each peak labeled with compound number. m/z calculated mono isotopic [M+2H]^2+^ for **17** C_67_H_108_N_18_O_18_S_2_ 758.3760 and oxidized form **18** C_67_H_108_N_18_O_18_S_2_ 758.3760.

Linear Dmaoc-protected SFTI-Dbz prepared by Fmoc SPPS was subjected to an intramolecular peptide bond formation followed by the removal of the Dmaoc group (Figure 6, Figures S-2,S-3). The sequential reactions in the conversion of Dbz to Bt and the subsequent coupling steps resulted in the formation of disulfide bonded cyclic SFTI(Dmaoc) **15** and a cyclic SFTI(Dmaoc)-NO adduct **16**. These products were afforded because the starting peptide **13** had free thiols, which could be oxidized by NaNO_2_. We expected that the NO group would be removed simultaneously during the deprotection of the Dmaoc group with thiol. The subsequent deprotection of Dmaoc using DTT successfully yielded the reduced form of cyclic SFTI **17** (43% isolated yield over three steps). This product was converted into the corresponding oxidized form of SFTI **18** quantitatively by treatment with DMSO (Figure 6C, Figure S-3). The synthesis of cyclic peptide SFTI demonstrated the utility of our peptide coupling strategy using the Dmaoc and Dbz groups for the chemical synthesis of peptide derivatives.

## Conclusion

We reported the Dmaoc as a polar protecting group and its use in peptide coupling reactions. The polarity of Dmaoc is higher than that of the Boc group, which is a conventional protecting group of the *N*^ε^-amines of Lys. The Dmaoc-protected peptides could be synthesized by standard Fmoc-SPPS straightforwardly and were successfully used for the synthesis of Sunflower trypsin inhibitor peptide. The Dmaoc group was stable during the synthesis but removable in a buffer containing thiol, thereby achieving the facile deprotection of Dmaoc after the peptide coupling reactions. The unique character of the Dmaoc group would expand the chemical toolbox for the synthesis of peptides and proteins, which would contribute to the development of next-generation drugs.

## Data Availability

All datasets generated for this study are included in the manuscript and/or the Supplementary Files.

## Author Contributions

All experiments and data analysis were carried out by RO and EO. The experiment design and manuscript preparation were done by RO. MI assisted with the experiment design. YK assisted with experiment design and the preparation of manuscript. All authors were involved in the discussion and have approved the submitted manuscript.

### Conflict of Interest Statement

The authors declare that the research was conducted in the absence of any commercial or financial relationships that could be construed as a potential conflict of interest.
