# Supplementary material for: N,N-Dimethylaminoxy Carbonyl, a Polar Protecting Group for Efficient Peptide Synthesis
Source: Front Chem. 2019 Mar 28;7:173. doi: 10.3389/fchem.2019.00173 (PMC6447706; doi:10.3389/fchem.2019.00173)
Supplement: Supplementary file 1 [file Data_Sheet_1.docx]

Supplementary Material

*N,N*-Dimethylaminoxy carbonyl, a polar protecting group for efficient peptide coupling reactions

Ryo Okamoto^1^*, Emiko Ono^1^, Masayuki Izumi^1^, Yasuhiro Kajihara^1^

^1^Department of Chemistry, Osaka University, Toyonaka, Osaka, JAPAN

*** Correspondence:**Ryo Okamoto
rokamoto@chem.sci.osaka-u.ac.jp

**Abbreviations**

DCM Dichloromethane

DIEA *N,N*-Diisopropylethylamine

DMF *N,N*-Dimethylformamide

HATU 1-[Bis(dimethylamino)methylene]-1*H*-1,2,3-triazolo[4,5-*b*]pyridinium 3-oxide hexafluorophosphate

HBTU 1-[Bis(dimethylamino)methylene]-1*H*-benzotriazolium 3-oxide hexafluorophosphate

HOOBt 3-Hydroxy-1,2,3-benzotriazine-4(3H)-one

FA Formic Acid

TFA Trifluoroacetic acid

EtOAc Ethyl acetate

DTT (±)-Dithiothreitol

ODS Octadecyl-functionalized silica gel

LCMS Liquid chromatography-mass spectrometry

SPPS Solid phase peptide synthesis

**General procedures**

Thin layer chromatography was performed using Silica gel 60 F_254_ (Merck TLC plates) and visualizations were performed with UV light (254 nm) and/or sulfuric acid stain (3 % H_2_SO_4_ in CH_3_OH). All reagents were purchased from commercial suppliers and were used without further purification. All the reaction solvents were dried over activated molecular sieves prior to use. Solvents were removed under reduced pressure at < 40 ^º^C. ^1^H and ^13^C NMR spectra were recorded on Bruker Avance III spectrometer (400 MHz for ^1^H, 100 MHz for ^13^C). All NMR signals were assigned on the basis of ^1^H NMR, COSY, HMBC and HSQC. HRMS was recorded on Q-TOF mass spectrometry by electron spray ionization (ESI) method.

**Analytical LCMS**

The purities and masses of synthesized Lys derivatives and peptides were confirmed by analytical LCMS as following. Chromatographic separations were performed by using a linear gradient of buffer B in buffer A over 15 or 20 min. Buffer A: 0.1 % FA in water, buffer B: 0.09 % FA in 90% acetonitrile and 10 % water. Proteonavi C4 silica gel with column dimension 2.0 x 150 mm was used for the analytical column. The eluent was monitoring by UV-absorbance at 218 nm for peptides and 280 nm for Fmoc-Lys derivatives, with on-line ESI-MS. Reported mass measurements are the sum of the ion currents across the major UV peak in each chromatogram.

**The synthesis of *N*^α^-(9-fluorenylmethyloxycarbonyl)-L-lysine *tert*-butyl ester (4)**

To a solution of Fmoc-Lys(Boc) **2** (6.0 g) in DCM (128 mL) was added *tert*-butyl 2,2,2-trichloroacetamidate (11.5 mL, 5.0 fold excess) and boron trifluoride ethyl ether complex (0.32 mL). This solution was stirred for 1.5 h in ice-salt bath (ca. -5 ℃) under Ar atmosphere. Then, the mixture was diluted with DCM, washed with sat. NH_4_Cl aq., H_2_O and brine. The organic phase was dried over MgSO_4_ and concentrated *in vacuo*. The crude Fmoc-Lys(Boc)-O*t*Bu **3** thus obtained was dissolved in dichloromethane (25 mL) chilled by an ice-salt bath. To the solution was added the solution of TFA (18 mL) and dichloromethane (25 mL). After stirring for 30 min, the solution was concentrated *in vacuo*. Purification of the residue by silica gel chromatography (EtOAc only → EtOAc : MeOH = 10:1) afforded compound **4** (3.1 g, 86 % over 2 steps) as a white solid. Analytical data: ^1^H(CD_3_CN): δ [ppm] = 7.82 (d, 2H), 7.67 (dd, 2H), 7.56 (br, 2H), 7.40 (t, 2H), 7.32 (t, 2H), 6.18 (d, 1H), 4.35-4.24 (m, 2H), 4.21 (t, 1H), 3.98 (m, 1H), 2.91 (br, 2H), 1.81-1.50 (s, 4H), 1.40 (2, 11H); ^13^C(CD_3_CN) δ 172.6, 157.2, 145.1, 145.0, 142.1, 128.7, 128.1, 126.2, 121.0, 82.2, 67.2, 55.5, 47.9, 40.3, 31.7, 28.1, 27.4, 23.2; HRMS (ESI-QTOF) *m/z*: [M+H]^+^ calcd for C_25_H_33_N_2_O_4_ 425.2435; Obsd 425.2407.

**The synthesis of *N*^α^-(9-fluorenylmethyloxycarbonyl)-*N*^ε^-(dimethylaminoxycarbonyl) -L-lysine *tert*-butyl ester (5)**

To the solution of **4** (10.9 g) in acetonitrile (256 mL) was added to *N,N*'-carbonyldiimidazole (4.57 g, 1.1 eq.). This mixture was stirred at room temperature under Ar for 1 h. Then, the reaction solution was added to *N,N*-dimethyl hydroxylamine hydrochloride (7.49 g, 3 eq.) and stirred for 10 h. The resultant mixture was diluted with EtOAc (2-3 times), extracted with sat. NH_4_Cl aq., water and brine (two times for each step), and dried over Na_2_SO_4_, filtered, and concentrated *in vacuo*. The crude residue was purified by flash column chromatography (EtOAc /hexane = 1:1 → 3:2) to give **5** (6.0 g, 46 %).　Analytical data: ^1^H (CDCl_3_): δ [ppm] = 7.77 (d, 2H), 7.61 (dd, 2H), 7.41 (t, 2H), 7.32 (t, 2H), 6.66 (br, 1H), 5.40 (d, 1H), 4.38 (m, 2H), 4.31-4.19 (m, 2H), 3.23 (dd, 2H), 2.71 (s, 6H), 1.86 (m, 1H), 1.75-1.31 (m, 14H); ^13^C(CDCl_3_) δ 171.5, 156.3, 155.9, 143.8, 143.7, 141.2, 127.7, 127.0, 125.1, 119.9, 82.2, 66.9, 54.0, 48.1, 47.1, 40.5, 32.4, 29.4, 27.9, 22.2; HRMS (ESI-QTOF) *m/z*: [M+H]^+^ calcd for C_28_H_38_N_3_O_6_ 512.2765; Obsd 512.2755.

**The synthesis of *N*^α^-(9-fluorenylmethyloxycarbonyl)-*N*^ε^-(dimethylaminoxycarbonyl) -L-lysine (1)**

Fmoc-Lys(Dmaoc)-O*t*Bu **5** (440 mg) was dissolved in 6 M HCl aq. /1,4-dioxane (1 : 1, 4.3 mL). The mixture was concentrated after 30 min. The solution was diluted with 1,4-dioxine and concentrated *in vacuo*. Purification of the residue by ODS column chromatography (100 % H_2_O→ H_2_O: acetonitrile = 80 % : 20 % →H_2_O: acetonitrile = 60 % : 40 %) afforded **1** (358 mg, 91%) after lyophilize. Analytical data: ^1^H(CD_3_CN) : δ [ppm] = 7.83 (d, 2H), 7.68 (dd, 2H), 7.42 (t, 2H), 7.34 (t, 2H), 6.90 (br, 1H), 6.04 (d, 1H), 4.32 (m, 2H), 4.23 (t, *1*H), 4.10 (m, 1H), 3.12 (dd, 2H), 2.66 (s, 6H), 1.80 (m, 1H), 1.68 (m, 1H), 1.53-1.32 (m, 4H); ^13^C(CD_3_CN) δ 174.5, 157.3, 145.3, 145.2, 142.2, 128.8, 128.2, 126.3, 121.1, 67.3, 55.0, 48.4, 48.1, 41.0, 31.9, 30.1, 23.6; HRMS (ESI-QTOF) m/z: [M+H]^+^ calcd for C_24_H_30_N_3_O_6_ 456.2129; Obsd 456.2124.

**Fmoc SPPS of Dmaoc-proteced peptides**

The synthesis of Dmaoc-protected peptides **6** and **13** were carried out on a 0.05 mmol of Dawson Dbz AM resin (Merk Millipore) by essentially same manner with the Fmoc SPPS protocol reported by Blanco-Canosa *et al* (*Angew. Chem. Int. Ed.* **2008,** 47, 6851) as following.

**The synthesis of peptide 6**

Fmoc-Pro-OH (0.2 mmol, 4 fold excess), HATU (0.2 mmol, 4 fold excess) and DIEA (52 μL, 0.3 mmol, 6 fold excess) were dissolved in DMF (1 mL). After 1 min pre-activation, this solution was added to Fmoc-Dawson resin and reacted for 1 h under gentle shaking (ca. 200 rpm). The following coupling was performed using 0.2 M HBTU/HOBt in DMF (975 μL), 0.2 M DIEA in DMF (1.5 mL, in DMF) and Fmoc-aa (4 fold excess to the resin used). Pre-activation was performed for 1 min prior to add to the resin and coupling was performed for 40 min. For the coupling of Fmoc-Lys(Dmaoc)-OH, 0.2 M HBTU/HOBt (1:1) in DMF (488 μL), 0.2 M DIEA in DMF (0.75 mL, in DMF) and Fmoc- Lys(Dmaoc)-OH (46 mg, 2 fold excess) were used. Removal of Fmoc group was performed by treatment with 20 % piperidine/DMF solution for 10 min in each step. After the completion of peptide chain assembly, the resin was washed with DMF and DCM. To the resin was added an acid cocktail containing TFA (1.9 mL), triisopropylsilane (50 μL) and H_2_O (50 μL) at ambient temperature. The mixture was gentely shaking for 60 min. The TFA solution containing the peptide was concentrated *in vacuo* to a minimal volume, added over cold ether and precipitated by centrifugation. The supernatant was removed and the residue was lyophilized. Purification of the resultant material by preparative LC (Proteonavi C4 10 × 250 mm, isocratic of 0.1 % TFA : 90 % CH_3_CN, 0.09 % TFA from 70:30 for 10 min followed by a linear gradient 0.1 % TFA : 90 % CH_3_CN, 0.09 % TFA from 70:30 to 20:80 over 60 min at a flow rate of 2.5 ml/min) afforded Dmaoc protected tetra-peptide **6** (29 mg, 66 % isolated). HRMS for **6** (ESI-QTOF) *m/z:* [M+H]^+^ calcd for C_45_H_60_N_9_O_9_ 870.4509; Obsd 870.4469.

**The synthesis of peptide 13**

The sythesis of SFTI peptide was also peformed by the essentially same manner with the synthesis of the tetrapeptide **6** afforded Dmaoc proteceted SFTI peptide **13** (10 mg, 11 % isolated for 0.05 mmol scale synthesis). Purification condition: Proteonavi C4 Φ10 × 250 mm, linear gradient 0.1 % TFA : 90 % CH_3_CN, 0.09 % TFA from 80:20 to 40:60 over 120 min at a flow rate of 2.5 ml/min). HRMS for **13** (ESI-QTOF) *m/z*: [M+H]^+^ calcd for C_77_H_121_N_22_O_21_S_2_ 1753.8513; Obsd 1753.8114.

**Coupling of peptide 6 and 8**

DMF (393 μL) and 4 M HCl in 1,4-dioxane (10 μL) were mixed and chilled in an ice bath. This acidic DMF solution was added NaNO_2_ (1.4 mg, 5 fold excess) and sonicated for 30 sec. The resultant suspension was chilled in an ice ethanol bath (ca. -17 ℃) and was added to the peptide **6** (3.5 mg, 4.0 μmol). After 5 min reaction under the same temperature, this reaction mixture was added to the peptide **8** dissolved in DMF (403 μL) containing HOOBt (6.6 mg, 10 fold excess) and DIEA (7.0 μL, 10 fold excess), and was left for 14 h at 0 ℃. The reaction mixture was subjected to ether precipitation by adding chilled ether (ca. 14 mL). After centrifugation of this crude suspension, the ether layer was removed by decantation to separate afforded precipitate. This ether-washing was repeated for 3 times. The resultant crude peptide **9** was dissolved in 0.1 M sodium phosphate buffer containing 6 M guanidine hydrochloride and 0.2 M DTT (pH 6.9, 806 μL) and reacted for 2 h at 37 ℃. To the solution was added piperidine (81 μL) and left for additional 40 min. After neutralization by adding 5 M HCl (186 μL), the reaction mixture was subjected to semi preparative RP-HPLC purification and afforded peptide **12** (2.8 mg, 60% isolated yield).

**Cyclization of peptide 13 (synthesis of SFTI)**

The solution of DMF (167 μL) and 4 M HCl/1,4-dioxane (4 μL) was added NaNO_2_ (0.6 mg, 5 fold excess) and sonicated for 30 sec followed by chilled in an ice-ethanol bath (ca. -15 ℃). The resultant suspension was added to the peptide **13** (3 mg, 1.7 μmol) and was left for 5 min in the same ice/ethanol bath (ca. -15 ℃). The mixture was subsequently added DMF (684 μL) containing HOOBt (2.8 mg, 10 fold excess) and DIEA (3.0 μL, 10 fold excess), then left for 30 min at 0 ℃. The reaction mixture was subjected to ether precipitation by adding chilled ether (ca. 14 mL). After centrifuge of this crude suspension, the afforded precipitate was separated. This step was repeated for 3 times. The resultant crude material was dissolved in 0.1 M sodium phosphate buffer containing 6 M guanidine hydrochloride and 0.2 M DTT (pH 7.0, 855 μL) and reacted for 3.5 h at 37 ℃. Purification of this reaction mixture by semi preparative RP-HPLC purification and afforded 1.3 mg of peptide **17** (43 % isolated yield).

**Oxidation of the cyclic SFTI 17**

Reduced form of SFTI **17** (1.3 mg) was dissolved in the mixed solution of 50 % CH_3_CN aq. (430 μL) and DMSO (107 μL). This mixture was left for 48 h under stirring at toom temperature, and then directly liophilized.


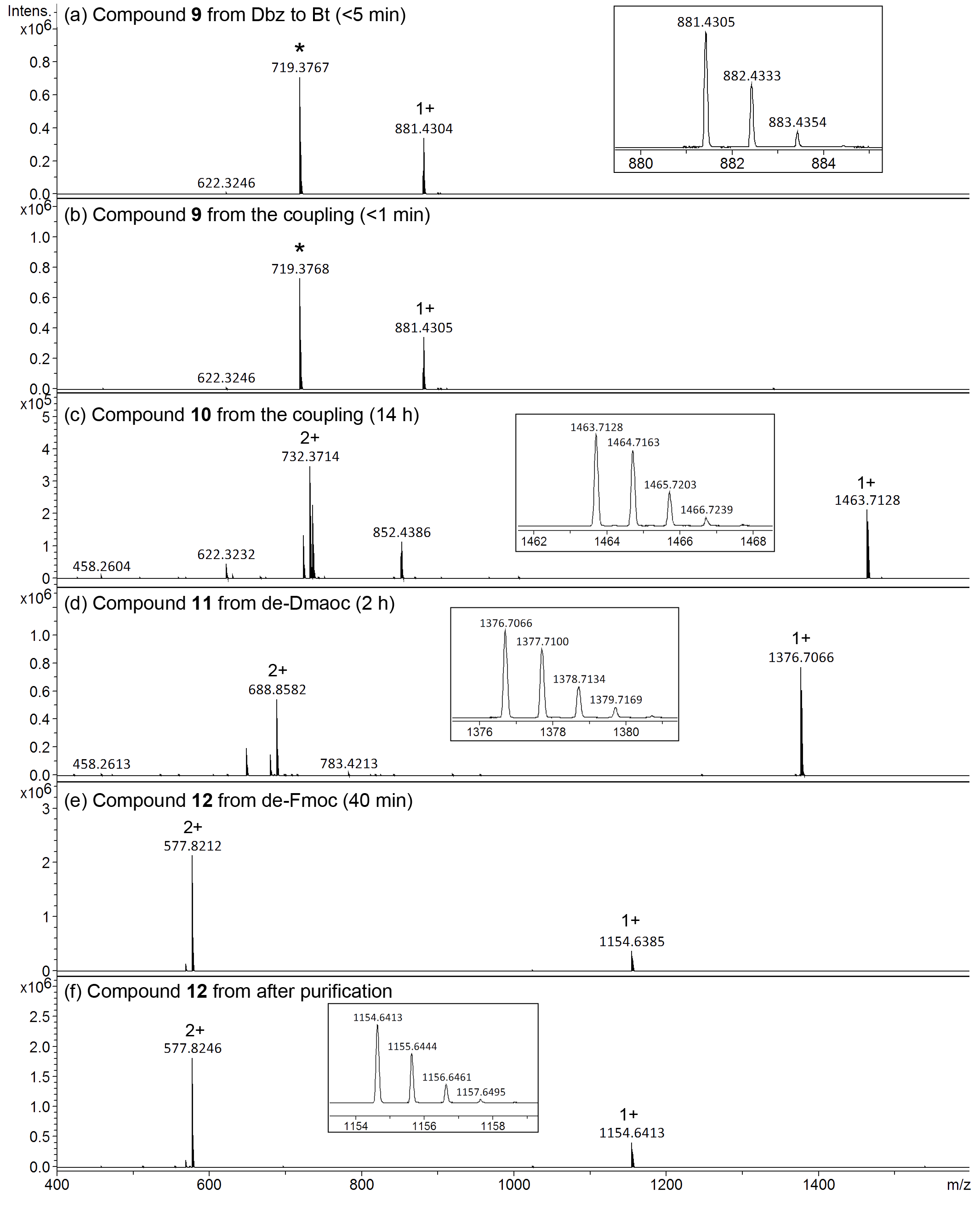


**Figure S-1**. HRMS spectra for the direct coupling of peptide **6** and **8.** The data were acquired from LC-MS analysis, of which the profile is shown in Figure 5 (main text). Peak labelded with asterisk is the hydrolized product of compound **9** plausibly generated in MS spectrometer. Insets: Expanded view of [M+H]^+^ ion peak of each spectrum. Calculated *m/z* (mono isotopic): [M+H]^+^ for compound **9** C_45_H_57_N_10_O_9_^+^ 881.4304; for compound **10** C_69_H_103_N_14_O_21_^+^ 1463.7417; for compound **11** C_66_H_98_N_13_O_19_^+^ 1376.7096; for compound **12** C_51_H_87_N_13_O_17_ 1154.6416


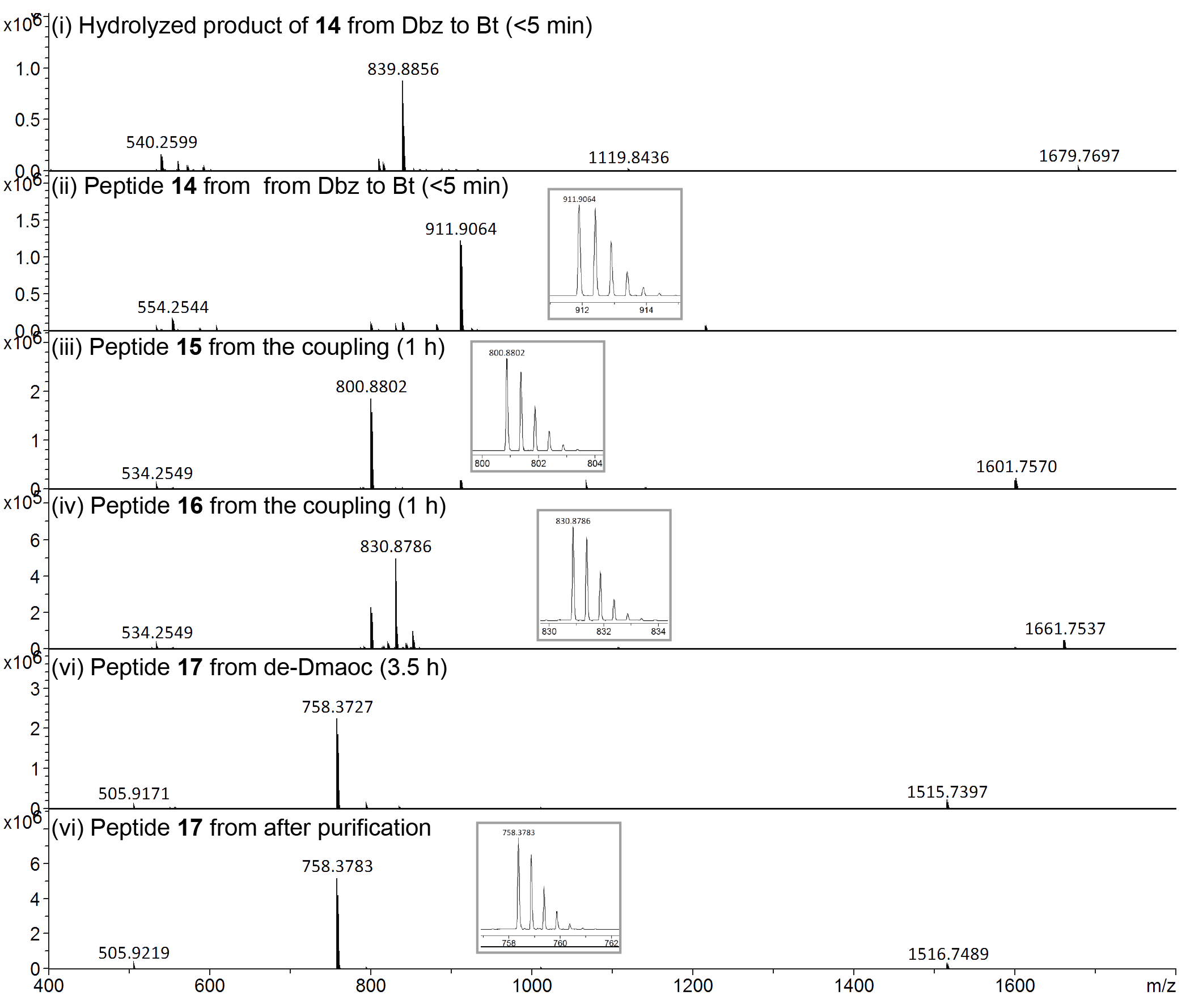


**Figure S-2.** HRMS spectra for the cyclization of peptide **13.** The data was acquired from LC-MS analysis, of which LC profile is shown in Figure 4 (main text). Hydrolyzed form of peptide **14** (spectrum (i) and Figure 4 (B-a)) was observed during the conversion of Dbz to Bt of peptide **13**. This was plausibly afforded during LC-MS analysis and not from reaction mixture, since this product was not pbserved in the following coupling step. Insets are expanded view of [M+2H]^2+^ ion peaks acquired by on-line ESI-HRMS from each peak labeled with compound number. Calculated *m/z* (mono isotopic): [M+2H]^2+^ for **14** C_77_H_117_N_25_O_23_S_2_ 911.9092; for **15** C_70_H_111_N_19_O_20_S_2_ 800.8841; for **16** C_70_H_111_N_21_O_22_S_2_ 830.8821; for **17** C_67_H_108_N_18_O_18_S_2_ 758.3760; Analytical condition: Linear gradient 0.1 % HCOOH aq.: 90 % acetonitrile aq. containing 0.09 % HCOOH from 90:10 to 30:70 over 15 min at a flow rate of 0.3 ml/min.

**
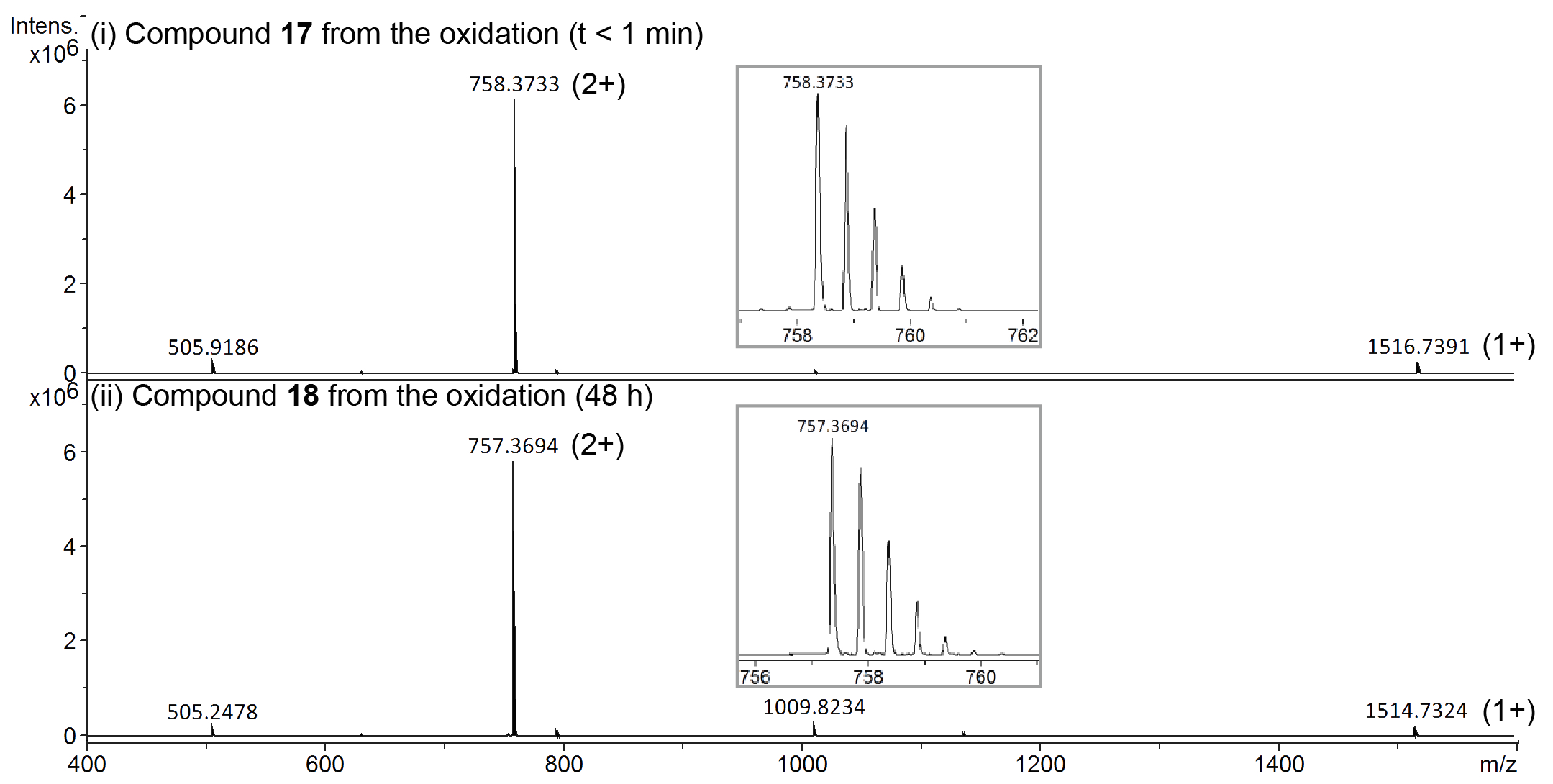
**

**Figure S-3**. HRMS spectra for the oxidation of SFTI **17**. Each spectrum was corresponding with LC peak shown in Figure 4 (main text). Insets are expanded view of [M+2H]^2+^ ion peak acquired by on-line ESI-HRMS from each peak labeled with compound number. Calculated *m/z* (mono isotopic): [M+2H]^2+^ for **17** C_67_H_108_N_18_O_18_S_2_ 758.3760 and for **18** C_67_H_106_N_18_O_18_S2 757.3681.

_
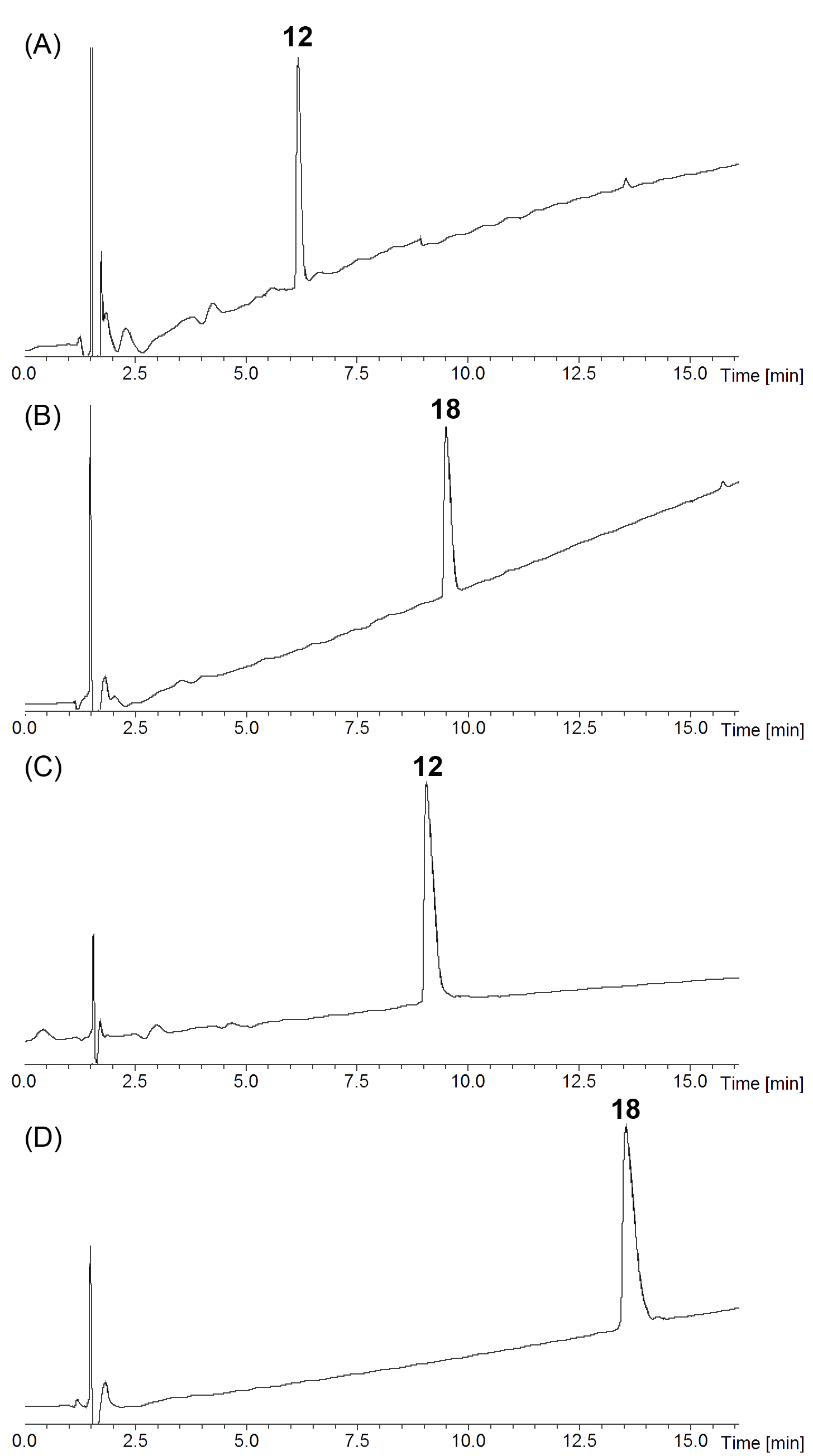
_

**Figure S-4**. RP-HPLC chromatograms for the peptide products **12** and **18**. The chromatographic separation was performed using linear gradient of solvent A : solvent B from 90:10 to 30:70 over 15 min at a flow rate of 0.3 ml/min; For solvent A and B, two different systems were used: 0.1 % HCOOH aq. / 90 % acetonitrile aq. containing 0.09 % HCOOH (condition **a**) and 0.1 % HCOOH aq. / 90 % methanol aq. containing 0.09 % HCOOH (condition **b**). (A) RP-HPLC chromatogram for peptide **12** using the condition **a**; (B) RP-HPLC chromatogram for peptide **12** using the condition **b**; (C) RP-HPLC chromatogram for peptide **18** using the condition **a**; (D) RP-HPLC chromatogram for peptide **18** using the condition **b.** The eluent was monitoring by UV-absorbance at 218 nm with on-line ESI-MS.
